# Quality of life and psychosocial risks in primary care workers in an urban area

**DOI:** 10.47626/1679-4435-2022-1031

**Published:** 2024-09-24

**Authors:** Miguel Andrez Valencia-Contrera, Sandra Verónica Valenzuela-Suazo, Carolina Elena Luengo-Martínez, María Olga Quintana-Zavala

**Affiliations:** 1 Programa de Magíster en Enfermería, Universidad de Concepción, Concepción, Región del Biobío, Chile; 2 Programa de Doctorado en Ciencia de Enfermería, Universidad Andrés Bello, Santiago, Región Metropolitana, Chile; 3 Facultad de Enfermería, Universidad Andrés Bello, Santiago, Chile; 4 Programa de Doctorado en Enfermería, Universidad de Concepción, Concepción, Región del Biobío, Chile; 5 Departamento de Enfermería, Universidad del Bío-Bío, Chillán, Región de Ñuble, Chile; 6 Departamento de Enfermería, Universidad de Sonora, Hermonsillo, Sonora, México

**Keywords:** psychosocial impact, primary health care, quality of life, coronavirus infections, occupational exposure, psychosocial factors, impacto psicosocial, atención primaria de salud, calidad de vida, infecciones por coronavirus, exposición profesional, riesgos psicosociales

## Abstract

**Introduction:**

Health workers are exposed to a wide variety of risks in their workplaces, including psychosocial risks, which are increasingly taking on special importance, with primary health care being little studied in this area, despite having taken great responsibilities in the COVID-19 pandemic.

**Objectives:**

To analyze the relationship between psychosocial risks and quality of life in health team workers in Family Health Centers Antofagasta, Chile, in 2021, in the context of the COVID-19 pandemic.

**Methods:**

This was a quantitative study, analytical, descriptive, cross-sectional type with 78 workers from the Primary Care health team of three Family Health Centers in the city of Antofagasta. The *Superintendência de Seguridad Social/Instituto Sindical de Trabajo, Ambiente y Salud* 21 brief version and the World Health Organization Quality of Life instrument-Abbreviated version instrument were applied.

**Results:**

The presence of a globally high psychosocial risk stands out, being this classified as high risk/level 1. The professional groups with the highest risks were nursing technicians and nurses; regarding quality of life, the dimension with the lowest score was psychological health, with a mean of 73.6.

**Conclusions:**

A negative relationship between psychosocial risks in the workplace and workers’ quality of life was evidenced In this hostile scenario, it is imperative that nurses, at a tactical and strategic level, promote workers’ health, cultivate healthy work environments, promote labor relations, and exercise more empathetic leadership as care managers.

## Abstract (pt)

**Introducción:**

Los trabajadores de la salud están expuestos a una gran variedad de riesgos en sus lugares de trabajos, incluyendo los riesgos psicosociales, que cada vez toman una especial importancia, siendo la atención primaria de la salud pobremente abordada en esta área, pese a que haya asumido grandes responsabilidades en la pandemia por COVID-19.

**Objetivos:**

Analizar la relación de los riesgos psicosociales y la calidad de vida en trabajadores del equipo de salud en Centros de Salud Familiar de Antofagasta, Chile, en 2021, en el contexto de la pandemia por COVID-19.

**Métodos:**

Se trata de un estudio de tipo cuantitativo, analítico, descriptivo, de corte transversal, con 78 trabajadores del equipo de salud de Atención Primaria de tres Centros de Salud Familiar de la ciudad de Antofagasta. Se aplicaron los instrumentos Superintendencia de Seguridad Social/Instituto Sindical de Trabajo, Ambiente y Salud 21 version breve y World Health Organization Quality of Life instrument-Abbreviated version.

**Resultados:**

Se destaca la presencia de un riesgo psicosocial elevado de manera global, siendo este clasificado como riesgo alto/nivel 1. Los estamentos con mayores riesgos fueron los técnicos de enfermería y enfermería; en lo referente a la calidad de vida, la dimension con menor puntaje fue la salud psicológica, con media de 73,6.

**Conclusiones:**

Se evidenció una relación negativa entre riesgos psicosociales en el lugar de trabajo y calidad de vida de los trabajadores. Ante este escenario hostil, se hace perentorio que los enfermeros, a nivel táctico y estratégico, promuevan la salud de los trabajadores, cultiven ambientes laborales saludables, favorezcan las relaciones laborales y ejerzan un liderazgo más empático como gestores del cuidado.

## INTRODUCTION

A recent report by the World Health Organization (WHO) and the International Labor Organization states that almost 2 million people die from work-related causes each year,^1^ since workers are exposed to a complex myriad of health and safety risks every day, including psychosocial risks,^2^ which have increasingly taken special relevance.^3^

Psychosocial risks were defined, in the context of the nineth meeting on occupational medicine held in Ginebra in 1984, as

interactions between work environment, work content, organizational conditions, workers’ capacities, needs, and culture, and personal considerations outside work that can, through perceptions and experience, influence health, work performance, and job satisfaction.^4^

At the end of 2019, a new type of virus causing a disease called COVID-19 spread rapidly until being considered a worldwide epidemic on March 11, 2020.^5^ In view of this hostile scenario, psychosocial risks may emerge or be exacerbated,^6^ both due the work and personal spheres, because the changes that the pandemic has brought can lead to exposure to several sources of psychosocial risks and therefore have a negative impact on workers’ quality of life.

In Chile, in order to respond to the pandemic, a health ministry protocol named Testing, Tracking, and Isolating (*Testeo, Trazabilidad y Aislamiento*) strategy has been implemented, which has been transversely developed by all levels of care, with emphasis on the key role of Primary Health Care (PHC) in the process, by means of interventions for timely detection of positive cases, tracking of close contacts, and transfer to health residences if necessary^7^; however, despite the important role played by PHC, there are scarce studies focused on measurement of psychosocial risks in this level of care,^8^ highlighting the importance of addressing this issue.

Based on the foregoing, the present study was developed to analyze the relationship between psychological risks and quality of life in health team workers at Family Health Centers (*Centros de Salud Familiar,* CESFAM) from Antofagasta, Chile, in 2021, during the COVID-19 pandemic.

## METHODS

This is a quantitative, analytical, descriptive, cross-sectional study^9^ conducted with 78 health care workers from three PHC centers in the city of Antofagasta selected through a non-probabilistic convenience sample method including the three largest PHC centers in the city, since these centers accounted for 70% of the population of interest. However, only 78 individuals signed the informed consent and comprised the final sample used.

Data collection took place from October to December 2021. Inclusion criterion was agreeing to voluntarily participate in the investigation by signing the informed consent. Exclusion criterion consisted of being on medical leave, vacations, or not performing work activities, for any reason, at the time of investigation.

Data collection was carried out by applying a total of 61 questions distributed into three questionnaires: the first of which named “bio-sociodemographic questionnaire,” created by the authors and including 15 questions to generate the workers’ profile (sex, age, marital status, children and relatives whom they take care of, educational level, disease diagnosed, professional group, time working in the position); the second questionnaire was the Superintendency of Social Security *(Superintendência de Seguridad Social,* SUSESO)/Union Institute of Work, Environment and Health (*Instituto Sindical de Trabajo, Ambiente y Salud,* ISTAS) 21 short version, consisting of 20 questions to measure the levels of workers’ psychosocial risks, a questionnaire validated for use in the Chilean population^10^; the third and last questionnaire was the WHO’s instrument named World Health Organization Quality of Life instrument-Abbreviated version (WHOQOL-Bref), consisting of 26 questions, used to measure participants’ quality of life and validated for use in the Chilean population.^11^

The investigation was approved by the Scientific Ethics Committee of Antofagasta Health Service, code CECSSA0008. After committee approval, the authors contacted the Antofagasta health directorate, which contacted each health center and authorized the investigation. Once approval from the institutions was obtained, each center director was requested to provide contact information from health care workers to offer participation in the study; each participant could accept or refuse participation in the study.

After data collection, information was stored on Microsoft Excel software, version 2011, on a personal computed to which only the primary author had access, who will keep this information for 5 years, in compliance with current national legislation in Chile. Subsequently, results were analyzed in the SPSS software version 25.0, applying descriptive statistics and Pearson’s coefficient.

## RESULTS

The final sample consisted of 78 workers, of which 86% were women; with regard to age, 47% were aged from 31 to 40 years, followed by 28% aged from 18 to 30 years, 17% from 41 to 50 years, 6% from 51 to 60 years, and finally 1% from 61 to 62 years. Table 1 presents the personal variables of the workers evaluated.

**Table 1:** Sociodemographic characterization of the Primary Health Care (PHC) team

Personal variables	Total (n = 78)	
	f	%
Marital status		
With a partner	53	67.94
Without a partner	25	32.05
Has children to take care of		
Yes	42	53.84
No	36	46.15
Number of children		
0	36	46.15
1	27	34.61
2	10	12.82
3	2	2.56
Does not specify number	3	3.84
Has relatives to take care of		
No	62	79.48
Yes, their mother	5	6.41
Yes, their parents	4	5.12
Yes, an uncle	1	1.28
Yes, but does not specify which relative is	5	6.41
Educational level		
Technical	23	29.48
Undergraduate	40	51.28
Specialization	8	10.25
Postgraduate	4	5.12
Postgraduate and specialization	3	3.84
Disease diagnosed		
None	45	5769
Hypothyroidism	5	6.41
Insulin resistance	7	8.97
Arterial hypertension	4	5.12
Other	17	21.79

*f* = absolute frequency

Table 2 describes work variables, such as profession, time working in the position, time of work experience, unit or service, type of employment contract, type of shift, and whether the participants work at another institution.

**Table 2:** Occupational characterization of Primary Health Care (PHC) workers

Work variables	Total (n = 78)	
	*f*	%
Professional group		
Undergraduate nursing technician	23	29.48
Dental technician	2	2.56
Medical technologist	1	1.28
Psychologist	2	2.56
Nutritionist	5	6.41
Midwife	5	6.41
Kinesiologist	8	10.25
Speech therapist	1	1.28
Nurse	13	16.66
Social worker	5	6.41
Pharmaceutical chemist	1	1.28
Physician	9	11.53
Dental surgeon	3	3.84
Time working in the position		
Less than 6 months	13	16.66
6 months to 1 year	7	8.97
1 year and 1 day to 3 years	13	16.66
3 years and 1 day or more	45	57.69
Time of work experience		
Less than 6 months	2	2.56
6 months to 1 year	4	5.12
1 year and 1 day to 3 years	11	14.10
3 years and 1 day or more	61	78.20
Unit or service		
Direct patient care	64	82.05
Administrative activity, warehouse, or other	14	17.94
Type of employment contract		
Honorary	18	23.07
Replacement	7	8.97
Fixed term	17	21.79
Indefinite	36	46.15
Type of shift		
Day	77	98.71
Four on four off	0	0.00
24 h	0	0.00
44 h per week	1	1.28
Works at another institution		
No	69	88.46
Yes	9	11.53

*f* = absolute frequency

**Figure 1:**
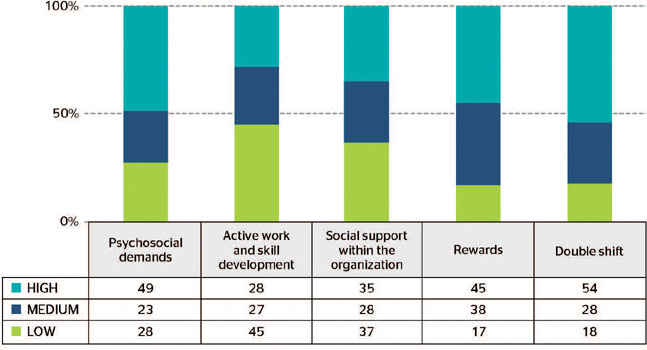
Prevalence of psychosocial risk level in Primary Health Care (PHC) workers.

The level of workers’ perception of psychosocial risk was classified as high/level 1 risk, since one dimension presented more than 50% of high risk, namely the double shift dimension. Figure 1 displays the five dimensions of the SUSESO/ISTAS 21 questionnaire and the percentage of high, medium, and low risks.

With regard to workers’ general quality of life, mean score was 3.7, with a minimum of 2 and a maximum of 5, and a standard deviation of 0.72. Table 3 shows quality of life results distributed according to WHOQOL-Bref dimensions.

**Table 3:** Quality of life of PHC workers, according to WHOQOL-Bref dimensions

WHOQOL-Bref domains	N	Mean	Median	Standard deviation	Minimum	Maximum
Physical health	78	74.46	77.14	11.41	45.71	97.14
Psychological health	78	73.63	73.33	13.16	46.66	100.00
Social relationships	78	74.95	80.00	17.59	20.00	100.00
Environment	78	74.90	77.50	11.04	45.00	95.00

PHC = Primary Health Care; WHOQOL-Bref = World Health Organization Quality of Life instrument-Abbreviated version.

In order to determine whether there is a relationship between each psychosocial risk dimension and each of the quality of life dimension, the Pearson’s correlation coefficient was applied, revealing a stronger negative correlation between the dimension “double shift” of psychosocial risks and with general quality of life, satisfaction with health status, with “physical health,” “psychological health,” and “environment” dimensions of the WHOQOL-Bref. This means that the higher the risks associated with “double shift,” the lower the quality of life of the workers evaluated, with the physical dimension of quality of life having the strongest relationship. Table 4 presents the results of correlations between the dimensions of each questionnaire.

**Table 4:** Correlation between psychosocial risks and quality of life in PHC workers

SUSESO-ISTAS 21 dimensions					
	Psychological demands	Active work and development of skills	Social support and quality of leadership	Rewards	Double shift
General quality of life	−0.033	−0.184	−0.103	−0.113	−0.297*
Satisfaction with health status	−0.097	−0.152	−0.139	−0.140	−0.316*
WHOQOL-Bref dimensions					
Physical health	−0.254*	−0.253*	−0.290*	−0.162	−0.444*
Psychological health	−0.057	−0.285*	−0.166	−0.229*	−0.226*
Social relationships	0.039	−0.207*	−0.040	−0.073	−0.137
Environment	−0.068	−0.159	−0.199	−0.279*	−0.411*

PHC = Primary Health Care; SUSESO-ISTAS = Superintendency of Social Security/Union Institute of Work, Environment and Health (*Superintendência de Seguridad Social/Instituto Sindical de Trabajo, Ambiente y Salud);* WHOQOL-Bref = World Health Organization Quality of Life instrument-Abbreviated version.

*Strongest negative correlations.

## DISCUSSION

With regard to bio-sociodemographic characteristics of the studied sample, most of which consisted of women, a result in agreement with findings observed in Chilean reports, as well as the predominance of individuals aged from 31 to 40 years.^12^

In relation to the variable “marital status,” most participants (nearly 68%) reported having a partner; it has been described that workers who have a partner and rely on their support have lower levels of family-work conflict.^13^ Conversely, when there are children, they were found to be an agent that causes family-work conflict,^14^ who etiology apparently lies on the stress experienced due to lack of time and energy, affecting mainly women, who are usually the main children’s caregiver; of the sample evaluated, nearly 54% reported taking care of at least one child, of which 35% took care of one child, 13% took care of two children, and 3% of three. Such stress associated with taking care of children may be reproduced or aggravated with the need of taking care of other relatives, a situation reported by 21% of the workers evaluated.

In turn, with regard to psychosocial risks, high levels, classified as high risk/level 1 risk, were identified in the sample evaluated, with values equal to or greater than 50% of high risk in one dimension of the SUSESO-ISTAS 21 questionnaire. The discouraging findings are in line with the reality observed by part of the scientific community, which described that the context of COVID-19 pandemic has brought a significant psychosocial burden on health care workers, although studies are limited in the PHC context.^8^ Conversely, evidence obtained prior to the COVID-19 pandemic found a hostile context in PHC, due to a more unfavorable psychosocial environment compared to the hospital setting.^15^

The double shift variable refers to concomitant demands in individuals’ work and family spheres; in the present investigation, it represents the dimension with the highest reported risk, accounting for 54% of workers who reported having a high risk, which may indicate that there are incompatible demands between the two domains. Such results are consistent with those described by Castro-Méndez,^16^ in a study that measured psychosocial risks in three Chilean PHC centers and showed that, in these centers, the dimension with the greatest risk was double shift; however, the investigation dates back from 2018, a period prior to the severe acute respiratory syndrome coronavirus 2 (SARS-CoV-2) pandemic; it is possible to assume that, as a result of the pandemic, this situation may have become worse.

In relation to the possible cause of risk posed by the double shift dimension, it is related to long working hours, or incompatible with personal or family life, strict schedules, and strict regulations for licenses or vacations.^17^ This aspect is closely related to the reality experienced in PHC, since the day shift that workers do is usually the one during which household activities are more required, especially in the context of the current sample, which is mainly composed of women, who have been historically responsible for reproductive work, also named “household” work.^18^ Although there have been advances in the journey towards equality between men and women, it is also true that, in this 21^st^ century, there is still a long way from achieving this goal.^19^

Concerning general quality of life and level of satisfaction with health status, the present investigation obtained scores classified as normal. A study developed in Brazil in 2007 with community health agents showed similar findings in the answers associated with quality of life,^20^ with a mean score of 3.98 and a standard deviation of 0.65. However, higher values were observed for satisfaction with health status, with a mean of 4.28 and a standard deviation of 0.72.

With regard to quality of life dimensions in the workers evaluated, the social relationships dimension stands out the one with highest scores. Such dimension includes sexual activity, personal relationships, and social support,^21^ the latter of which is considered, in the theoretical framework to approach psychosocial factors proposed by Ceballos-Vásquez et al.,^22^ as a protective factor that, in the concomitant presence of a positive perception of the work environment, may give rise to active work, low stress, high rewards, great esteem, and appropriate mental workload, thus favoring workers’ health, which may explain the reason why this is the dimension with the lowest risks in the sample. However, the psychological health dimension had the lowest score among the workers evaluated.

A Brazilian study^23^ also found the highest score for the social relationship dimension, with a mean of 75.8, although the one with the lowest score was environment, with 54.1, which is consistent with results obtained in two other Brazilian studies, whose means were 59.5^20^ and 58.0, respectively.^24^ This finding of lower scores for the environment dimension in the compared studies,^20,23,24^ was shown to be associated with workers’ unsafety resulting from exposure to urban violence without protection against this reality, which was present in several regions^25^; therefore, results are expected to differ from those of the present study.

Teles et al.^26^ described a significant association between effort-reward imbalance and poor quality of life, both for general quality of life and for the physical, social and environmental domains, consistent with the results of the present study, which found a negative relationship of -0.279 between the environment dimension of quality of life and the reward dimension, since the latter is associated with the recognition received by workers for their efforts, which may be closely related to reward dimension of the effort-reward imbalance model.^27^

Furthermore, a negative relationship of −0.254 was found between the psychological demands dimension and the physical health dimension of quality of life, consistent with the results described by Asante et al.,^28^ which found that workers have a highly demanding and strained working environment, with an impact on their quality of life. It is pertinent to consider that the current study is framed within the context of one of the most important public health problems in the 21^st^ century, i.e., the COVID-19 pandemic.^5^

In turn, values of -0.285 in the psychological health dimension and of -0.207 in the social relationship dimension are associated with the active work and skill development dimension. This is in line with findings described by Bustamante et al.,^12^ who report that recreation and family, support and institutional recognition, safety, work well-being, and development and integration affect perception of quality of work life.

An element that holds special importance in the present study is the urgent need of developing investigations in PHC, because this level of care is shown to play the leading role in the main strategies to fight against COVID-19. However, it is still perceived as the little sibling of the healthcare system, an analogy that fully reflects the need for its strengthening, which is explicit in a recent report by the Organisation for Economic Co-operation and Development that highlights the importance of investing in improving PHC services.^29^

The limitations of the present study are associated with sample size, because only 78 workers from the three PHC centers signed the free informed consent, out of a population of 350 workers distributed into eight PHC centers in the city of Antofagasta, which may be associated with the very tiredness from the activities performed in these spaces.

## CONCLUSIONS

The present study complied with the objective proposed, concluding that, in the evaluated sample of 78 PHC workers from three CESFAM in the city of Antofagasta, there was a negative or inverse relationship between psychosocial risks and health care workers’ quality of life, a result in line with the findings presented in the scarce scientific evidence reported so far. The psychosocial risk dimension named “double shift” stands out as the dimension with the strongest negative relationship with quality of life dimensions.
